# Genome Annotation of Poly(lactic acid) Degrading *Pseudomonas aeruginosa*, *Sphingobacterium* sp. and *Geobacillus* sp.

**DOI:** 10.3390/ijms22147385

**Published:** 2021-07-10

**Authors:** Sadia Mehmood Satti, Edgar Castro-Aguirre, Aamer Ali Shah, Terence L. Marsh, Rafael Auras

**Affiliations:** 1 Department of Microbiology, Faculty of Biological Sciences, Quaid-i-Azam University, Islamabad 45320, Pakistan; sadiraja_19@yahoo.com (S.M.S.); alishah@qau.edu.pk (A.A.S.); 2 School of Packaging, Michigan State University, East Lansing, MI 48824-1223, USA; ragde.187@gmail.com; 3 University Institute of Biochemistry and Biotechnology, PMAS Arid Agriculture University, Shamasabad, Muree Road, Rawalpindi 46300, Pakistan; 4 Kraft Heinz Company, Glenview, IL 60025-4312, USA; 5 Department of Microbiology and Molecular Genetics, Michigan State University, East Lansing, MI 48824-1223, USA

**Keywords:** genome sequence, poly(lactic acid) biodegradation, biofilm, hydrolytic enzymes

## Abstract

*Pseudomonas aeruginosa* and *Sphingobacterium* sp. are well known for their ability to decontaminate many environmental pollutants while *Geobacillus* sp. have been exploited for their thermostable enzymes. This study reports the annotation of genomes of *P. aeruginosa S3*, *Sphingobacterium S2* and *Geobacillus* EC-3 that were isolated from compost, based on their ability to degrade poly(lactic acid), PLA. Draft genomes of the strains were assembled from Illumina reads, annotated and viewed with the aim of gaining insight into the genetic elements involved in degradation of PLA. The draft genome of *Sphinogobacterium* strain S2 (435 contigs) was estimated at 5,604,691 bp and the draft genome of *P. aeruginosa* strain S3 (303 contigs) was estimated at 6,631,638 bp. The draft genome of the thermophile *Geobacillus* strain EC-3 (111 contigs) was estimated at 3,397,712 bp. A total of 5385 (60% with annotation), 6437 (80% with annotation) and 3790 (74% with annotation) protein-coding genes were predicted for strains S2, S3 and EC-3, respectively. Catabolic genes for the biodegradation of xenobiotics, aromatic compounds and lactic acid as well as the genes attributable to the establishment and regulation of biofilm were identified in all three draft genomes. Our results reveal essential genetic elements that facilitate PLA metabolism at mesophilic and thermophilic temperatures in these three isolates.

## 1. Introduction

The lactic acid required to produce poly(lactic acid) (PLA) through polycondensation is derived primarily from the microbial fermentation of agro-industrial waste. Frequently, waste by-products from crops like casava, wheat, bran, corn, etc., serve as the fermentable substrates [1,2]. PLA is certified as completely biodegradable under industrial composting conditions [3]. In the last two decades, the biodegradation of PLA has been extensively studied and many microbial species (actinomycete, bacteria, fungus) have been identified with the ability to degrade PLA [4]. Most of the reported bacterial species are from the families *Pseudonocardiaceae*, *Thermomonosporaceae*, *Micromonosporaceae*, *Streptosporangiaceae*, *Bacillaceae* and *Thermoactinomycetaceae* while the fungal species are mainly from the phyla Basidiomycota (*Tremellaceae*) and Ascomycota (*Trichocomaceae*, *Hypocreaceae*) [5,6,7,8,9,10,11].

In our previous study on mesophilic strains, we described four bacterial isolates designated as S1, S2, S3 and S4, able to degrade PLA at ambient temperature [12]. Two of the isolated strains, *Sphingobacterium* sp. (S2) and *P. aeruginosa* (S3), were evaluated for their PLA degradation in soil microcosms [13] and are the subject of this study. The genus *Sphingobacterium* is from the phylum Bacteriodetes, family *Sphingobacteriaceae*, named with reference to the sphingolipids in their cell wall [14,15]. They are Gram-negative rods, and the GC content of their DNA usually ranges from 35 to 44 mol% [16,17]. *Sphingobacterium* sp. are found in a range of habitats like soil, forest, compost, activated sludge, rhizosphere, feces, lakes and various food sources [18]. *Sphingobacterium* have also been reported to have a potential role in the biodegradation of different pollutants, including mixed plastic waste, PAHs, oils and pesticides [19,20,21]. *Pseudomonas aeruginosa* are Gram-negative bacteria from the γ-subdivision of proteobacteria. They are ubiquitously distributed in soil and aquatic habitats and are well-known opportunistic pathogens [22,23]. They have the ability to thrive in highly diverse and unusual ecological niches with low levels of nutrients. Their metabolic versatility and resistances allow them to survive on a variety of diverse carbon sources and overcome disinfectants and some antibiotics [24,25]. In previous reports, the role of *Pseudomonas aeruginosa* in the degradation of a variety of different compounds, including polycyclic aromatic hydrocarbons, xenobiotic compounds, oils, dyes and plastics is well documented [26,27,28,29,30]. In a second study we described the isolation of a thermophilic *Geobacillus* strain isolated with PLA as the sole source of carbon and capable of forming biofilm [31]. *Geobacillus* sp. are well-described Gram-positive spore forming rods whose thermophilic attributes have been exploited for highly stable enzymatic activities [32,33].

The five PLA degrading bacterial strains reported in our previous studies were isolated from compost and had the ability to degrade PLA at either ambient temperature, in the cases of *P. aeruginosa* and *Sphingomonas* sp., [12] or at elevated temperatures in the case of *Geobacillus* sp. [31]. Interestingly, our *P. aeruginosa* strains (S3 and S4) were lactate utilizing while the *Sphingobacterium* S2 and *Chryseobacterium* S1 strains were unable to utilize lactate when provided as the sole carbon source in minimal media. We also observed that all four isolates could form biofilm on PLA.

The purpose of this study was to analyze the genomes of three isolates (S2, S3 and EC-3) and explore the genetic determinants responsible for conferring their degradation abilities. The genes controlling lactate utilization and biofilm formation and regulation were inferred through comparative sequence analysis at PATRIC [34]. The whole genome sequence analysis for *P. aeruginosa* sp. is extensive but such data for *Sphingobacterium* sp. and *Geobacillus* sp. are limited. To our knowledge this is one of the first reports to give genetic depth to PLA-degrading bacterial strains and provide the basis for further studies.

## 2. Results

### 2.1. General Genome Features of Sphingobacterium sp. (S2), P. aeruginosa (S3) and Geobacillus sp. (EC-3)

Table 1 shows the general features for the assembled genomes. The assembly of the draft genome for strain S2 yielded 87 contigs (434,971 maximum length) and a total of 5,445,390 assembled base pairs with 43.66% GC content. There were an estimated 4951 CDS regions, 2864 proteins with functional assignments and several antibiotic resistance genes. The assembled draft genome of S3 had 63 contigs (658,980 maximum length) with a total of 6,509,961 assembled base pairs and was 66.26% GC content. There were an estimated 6239 CDS regions and 4932 proteins with functional assignments and a substantial number of putative antibiotic resistant and virulence determinants. As a thermophile, *Geobacillus* strain EC-3 had a smaller genome with 111 contigs and a total of 3,397,712 assembled base pairs with the largest contig of 321,190 bp. The G+C content was 52.18% with an estimated 3790 CDS regions and 2806 proteins with functional assignments. Because these were draft sequences, i.e., not closed, the number of chromosomes and plasmids could not be determined.

### 2.2. Genetic Relatedness Based on ANI

**Figure 1:**
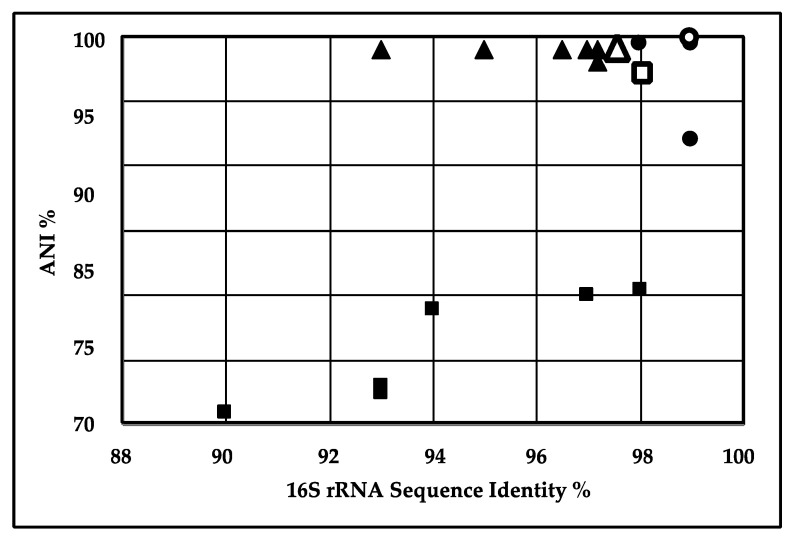
Correlation between 16S rRNA gene similarity and average nucleotide identity (ANI) of sequenced strains. *Sphingobacterium* S2 (∎), *P. aeruginosa* (▲) and *Geobacillus* EC-3 (●) were compared to their closest relatives by 16S rRNA similarity (abscissa) and ANI (ordinate). The closest relatives, *S. thalpophilum* DSM11723 (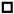), *P. aeruginosa* PSE305 (**△**) and *G. thermoleovorans* CCB_US3_UF5 (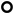), were used in the MAUVE alignments below. Note that the 16S rRNA sequences came from prior sequencing of the 16S rRNA gene [12,31].

The average nucleotide identity (ANI) value describes the similarity between the sequences of the conserved regions of two genomes and measures the genetic relatedness between them [35]. ANI measurements are considered more informative over 16S rRNA gene identity as they are based on a larger number of genes [36] and ANI values ≥ 95% should be considered as the same species [37]. ANI comparisons were done to explore the interspecies genetic relatedness between our three isolates and related species in the public databases. The data point in the upper-right corner of Figure 1 represents *S. thalpophilum* DSM11723 and *S. thalpophilum NCTC11429*, both of which showed more than 98% 16S rRNA gene similarity and 98% ANI with strain S2. Strain NCTC11429, isolated from a human wound (NCBI Biosample SAMEA3643323), was the closest match to our strain.

In the case of *P. aeruginosa* strain S3, Figure 1 shows a cluster in the upper-right corner containing closely related strains with more than 98–99% sequence similarity, based on 16S rRNA sequences and 93–97% ANI. *P. aeruginosa* PSE305 showed the highest ANI value and 16S rRNA gene identity of 97.69% and 99% respectively. Accordingly, *P. aeruginosa* strain S3 and other strains included in the analysis belong to the same species. The results are consistent with the phylogenetic results based on 16S rRNA gene identity as previously reported [12], but the ANI analysis indicated that our strain was closest to *P. aeruginosa* PSE305, rather than *P. aeruginosa* BUP2. Based on 16S rRNA comparative sequence analysis *P. aeruginosa* O12 PA7 showed 99% sequence similarity to *P. aeruginosa* strain S3 but had <95% ANI value. For *Geobacillus* sp. EC-3, Figure 1 shows a cluster in the upper-right corner containing closely related strains with more than 97% sequence similarity based on both 16S rRNA sequences and ANI analysis. The closest completed *Geobacillus* in the public databases was *Geobacillus thermoleovorans* strain CCB_US3_UF5. The proximity of *Geobacillus* sp. EC-3 to G. *thermoleovorans* (99.4% ANI and 99.8% 16S rRNA) suggests that EC-3 is a *thermoleovorans*. *Sphingobacterium* sp. strain S2 and *P. aeruginosa* strain S3 were isolated from the compost samples, while the most closely related strains, *S. thalpophilum* DSM11723 and NCTC11429 and *P. aeruginosa* PSE305, were isolated from human clinical samples. While *Pseudomonas aeruginosa* is a well-documented pathogen, even *Spingobacterium* has been identified as a potential opportunistic pathogen [38,39]. *Geobacillus* EC-3 was also isolated from compost while the closest strain in the database, G. *thermoleovorans* strain CCB_US3_UF5, was isolated from Ulu hot springs in Malaysia [40].

### 2.3. MAUVE and MeDuSa Alignments

**Figure 2:**
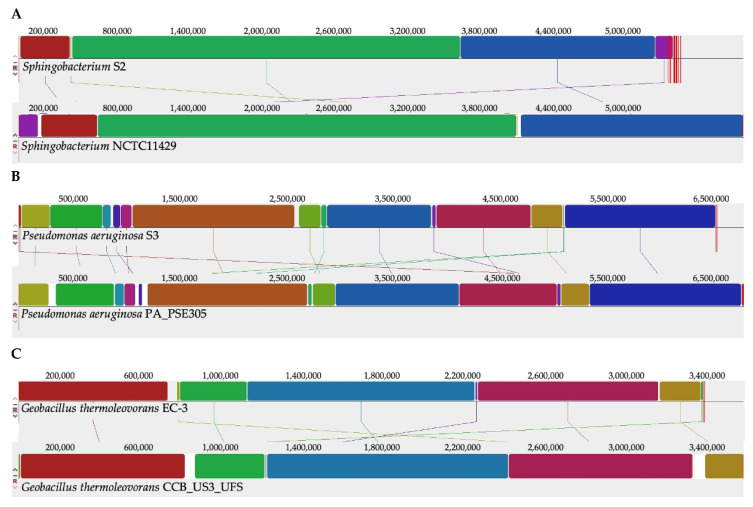
Genomic alignments generated in MeDuSa and MAUVE of *Sphingobacterium* sp. S2 with *Sphingbacterium thalpophilum* NCTC11429 (Panel **A**), *P. aeruginosa* S3 with *P. aeruginosa* PSE305 (Panel **B**) and *Geobacillus* EC-3 with *G. thermoleovorans* CCB_US3_UFS (Panel **C**). Similarly colored blocks indicate assembled regions of two genomes that are highly homologous. Note that the level of homology can vary across the blocks. These blocks are separated by white blocks representing regions with low or no homology. Genome size is indicated as number of base pairs.

MAUVE and MeDuSa were used to find sequence homologies of the two isolates with the strains identified as closest in the database by ANI calculations. The alignments shown in Figure 2 were derived by first aligning the SPADE-assembled contigs against reference strains, (*S. thalpophilum* NCTC11429, *P. aeruginosa* PSE305 and *G. thermoleovorans* strain CCB_US3_UF5) with MeDuSa [41], followed by alignment in MAUVE [42] using the MeDuSa ordered scaffolds of the isolates.

The MeDuSa alignment of contigs from the assembled *Sphingobacterium* S2 against *S*. *thalpophilum* NCTC11429 reduced the contig count from 87 to 26. *P. aeruginosa* PSE305 was used as a reference strain in MeDuSa which reduced the contig count from 63 to 9. MeDuSa reduced the 111 contigs of the Spades assembled *Geobacillus* sp. EC-3 to 4. Overall, the genomes of these isolates had good synteny with their closest relatives as measured by genome assembly and alignments in MeDuSa and MAUVE. Of particular interest was the assembly and alignments of *Geobacillus* sp. EC-3. EC-3 appears closest to *G. thermoleovorans* strain CCB_US3_UF5. However, there are several recent reports of *Geobacillus* genome projects and at least three isolates, strains ARTRW1, FJAT2391 and KCTC 3570, have a very similar genome organization, as evaluated in MAUVE, in comparison with EC-3 and CCB_US3_UF5, although they were isolated from fairly diverse sources. Strain ARTRW1 was isolated from hot springs in Turkey [43], strain FJAT2391 came from soil in China (Genbank Accession # PRJNA340206), and strain KCTC 3570 (Genbank Accession # PRJNA310809) came from soil near a hot effluent stream in Pennsylvania, USA. The alignment of the genomes of these strains is presented in Figure S1 (Supplemental Information). This conservation of genomic architecture suggests strong selective pressure.

### 2.4. Genetic Systems and Metabolism

**Figure 3:**
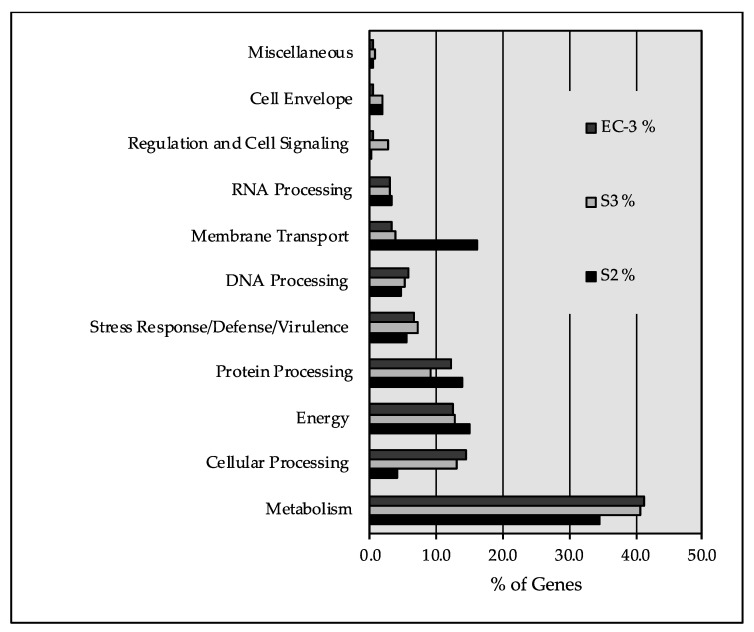
Distribution of identified genes to subsystems.

The distribution of identified genes to cell subsystems is shown in Figure 3. The distribution patterns are roughly the same between strains with the exception that a greater percentage of the genomes of S3 and EC-3 are devoted to “Cell Processing” compared to S2, whereas S2 has a greater genetic commitment to “Membrane Transport”.

**Figure 4:**
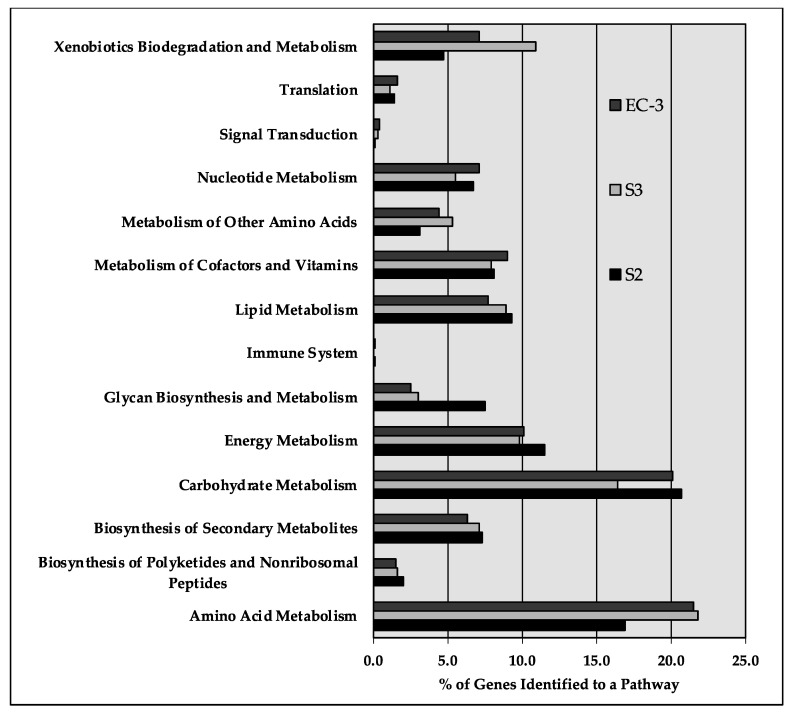
Distribution of genes to pathway categories in *Sphingobacterium* sp. S2, *P. aeruginosa* S3 and *Geobacillus* sp. EC-3. These data were derived from the pathways evaluation program as implemented in PATRIC [34].

*Sphingobacterium* spp. are Gram-negative rods, aerobic, exhibiting sliding motility and form yellow-pigmented colonies. *Sphingobacterium* have been isolated from diverse environments such as soil, water, compost, deserts, blood and urine samples from human patients. A distinctive feature of *Sphingobacterium* is the presence of sphingolipids at relatively high concentrations in their cell wall [15,44]. In *Sphingobacteria* S2, 1589 ORFs were assigned metabolic functions. Genes (269) were identified as participating in amino acid metabolism while 329 genes were dedicated to carbohydrate metabolism. Energy metabolism and lipid metabolism had 183 and 149 genes identified, respectively, while 76 genes were assigned to xenobiotic biodegradation and metabolism (Figure 4).

*P. aeruginosa* is a Gram-negative bacterium able to grow, aerobically, on a wide range of substrates and anaerobically, when nitrate is available as terminal electron acceptor. It is capable of thriving in highly diverse and unusual ecological niches with low availability of nutrients. Its metabolic versatility allows it to use a variety of diverse carbon sources, including certain disinfectants [45]. Moreover, it can synthesize a number of antimicrobial compounds [24,25]. Using genome annotation through PATRIC, 1084 ORFs in *P. aeruginosa* were assigned to metabolic pathways. Among the major pathways, 532 genes were assigned to amino acid metabolism and 241, 401, 194 and 219 genes were assigned for energy metabolism, carbohydrate metabolism, metabolism of cofactors and vitamins and lipid metabolism, respectively. Genes, 266, were identified as involved with xenobiotics while 136 and 174 genes were linked to nucleotide metabolism and biosynthesis of secondary metabolites.

*Geobacillus* spp. are Gram-positive spore forming rods that are found widely distributed. They are capable thermophiles and have been routinely isolated from hot springs and compost [40,46]. In strain EC-3, 1662 ORFs were assigned to metabolic functions with 357 devoted to amino acid metabolism, 334 assigned to carbohydrate metabolism, and 169 and 129 assigned to energy and lipid metabolism, respectively. While there are no glaring differences in gene distributions between the three genomes, it is, however, curious to note that *Sphingobacterium* S2 devotes twice as much genetic power to glycan biosynthesis and metabolism. This indicates that *Sphingobacterium* can express various enzymes involved in the synthesis and degradation of glycans that may be of value for various biotechnological applications.

### 2.5. Xenobiotic Biodegradation Metabolism

The role of *P. aeruginosa* in the degradation of large complex molecules including PAHs, xenobiotic compounds, oil, dyes and plastics is well documented [26,27,28,29,30]. *Sphingobacterium* spp. have also been reported to have a potential role in the biodegradation of different pollutants, including mixed plastic waste, PAHs, biodegradation of oil and pesticides [19,20,21]. *Geobacillus* spp. have been identified that can reduce azo dyes and degrade alpha-naphthol and nylon [47,48,49]. Table 2 describes the major pathways and the number of genes related to the biodegradation of different xenobiotic compounds in strains S2, S3 and EC-3. According to sequence analysis, numerous pathways for the biodegradation of xenobiotic compounds were found in the three strains with approximately three times as many in *P. aeruginosa S3* as compared to *Sphingobacterium* sp. S2 and *Geobacillus* sp. *EC-3.* This may be due, in part, to the fact that *P. aeruginosa* is more comprehensively studied compared to *Sphingobacterium* and *Geobacillus*. Among all the degradation pathways found in the three strains, the degradation of benzoate in *P. aeruginosa* had the greatest number of annotated genes. Benzoate, an aromatic compound, has been widely used as a model for the study of the bacterial catabolism of aromatic compounds [50]. Genetic evidence for the ability to degrade 1,4-dichlorobenzene, 2,4-dichlorobenzene and benzoate were detected in all three genomes. Many genes related to the degradation pathway of one of the most important classes of pollutants, PAHs, such as naphthalene, anthracene, 1- and 2-methylnaphthalene, were found in all three strains. Among the halogenated organic compounds, 29 genes were dedicated to tetrachloroethene degradation in *P. aeruginosa S3* while only 6 and 8 were found in *Sphingobacterium* sp. S2 and *Geobacillus* sp. EC-3, respectively. For aromatic compounds and chlorinated aromatic compounds, pathways for the biodegradation of toluene, trinitrotoluene, xylene degradation, 1,4-Dichlorobenzene degradation and 2,4-Dichlorobenzoate were also found. Genes for the biodegradation of Bisphenol A, one of the most abundantly produced chemicals released into the environment and a serious health concern and environmental pollutant, was also found in *P. aeruginosa* S3 [51] and *Sphingobacterium* sp. S2.

### 2.6. Lactate Metabolism

Lactate utilization as the sole carbon source is a property of many bacteria where a key step of the process is the oxidation of lactate [52,53,54,55,56]. Lactate dehydrogenases found in microbes are of two types, NAD-dependent lactate dehydrogenases (nLDHs) and NAD-independent lactate dehydrogenases (iLDHs), also called respiratory lactate. The latter is usually considered to be the enzyme mainly responsible for the metabolism of lactate as a carbon source [53]. The lactate utilization system is comprised of three main membrane bound proteins: NAD-independent lactate dehydrogenase (l-iLDH), NAD-independent D-lactate dehydrogenase (d-iLDH), and a lactate permease (LldP). Lactate permease is responsible for the uptake of lactate into the cells and lactate dehydrogenases carry out the oxidation of lactate to pyruvate [55,57]. Lactate utilization has been observed in some pathogens [55], stimulating their growth during infections while enhancing the synthesis of pathogenic determinants and increasing resistance against various bactericidal mechanisms [55]. The utilization of lactate by different *Pseudomonas* strains is well documented [56,58,59,60]. In the sequence analysis of our *P. aeruginosa* strain S3, a complete cascade of genes was found encoding the machinery for lactate utilization including a lactate permease, both l and d-lactate dehydrogenases and a lactate-responsive regulator LldR (Table 3). This strain was isolated and characterized for its potential to degrade poly(lactic acid) and its ability to utilize lactate as a sole carbon source was established in our previous study [12]. Presence of the lactate utilization machinery found through genome sequencing is consistent with previous observations that both l-iLDH and d-iLDH are present in the single operon and are induced coordinately in *Pseudomonas* strains and expression of both enzymes is controlled by the presence of an enantiomer of lactate [59].

In the genome analysis of our isolate *Sphingobacterium* sp. S2, we found an incomplete set of genes for lactate metabolism. Both l-lactate dehydrogenase and d-lactate dehydrogenase were present, but no lactate permease was detected (Table 3). Absence of lactate permease is consistent with our previous findings that showed that *Sphingobacterium* sp. S2 did not utilize lactic acid as the sole source of carbon. Inability to grow on lactic acid had been previously reported in literature for different strains of *Sphingobacterium* [18,61]. The fact that these strains were isolated, based on their ability to degrade PLA, suggests that a degradation product other than lactate is involved.

*Geobacillus* sp. EC-3 was capable of growing on lactate as the sole carbon source and we detected lactate dehydrogenase and lactate permease in strain EC-3. In addition, we identified lactate utilization proteins LutA, LutB and LutC, along with the lactate responsive regulator LutR. These lactate utilization proteins are discussed below, relative to biofilm formation.

### 2.7. Genetic Determinants for Biofilm Formation and Regulation

In contrast to the planktonic lifestyle, cells within a biofilm matrix are in close proximity where secreted enzymes provide optimal returns for the population, especially when targeting the substratum on which the biofilm forms [62]. The phenomenon of microbial biofilm formation is also related to other survival strategies like metal and antimicrobial resistance, tolerance and bioremediation [63,64]. The application of biofilm mediated bioremediation has been found superior to other bioremediation strategies and is being applied in bioremediation of different environmental pollutants [65,66,67,68,69]. Microorganisms that develop a biofilm through attachment and formation of an extracellular protective matrix are physiologically more resilient to environmental changes, making them a logical choice for the remediation of different pollutants. These microbes use different strategies like biosorption, bioaccumulation and biomineralization to slowly degrade compounds [70]. All three of our isolates were capable of forming biofilm on PLA. This was tested because the formation of biofilm on solid polymers that can be degraded for carbon and/or energy is a logical strategy for microbes. Excreted enzymes capable of degradation can be sequestered within the biofilm matrix in close proximity to the polymer and not lost to solution. Therefore, we sought to identify genes involved in biofilm formation in strains S2, S3 and EC-3. *P. aeruginosa* is a remarkably adept opportunist with striking ability to develop biofilm [22,71,72]. In our previous study, we also observed biofilm formation by our isolate *P. aeruginosa* strain S3 on the surface of PLA during the process of biodegradation [12]. The phenomenon of biofilm formation on the surface of PLA had previously been reported by other authors as well [37,73,74]. In the genetic analysis of our isolate, we found factors involved in the development of the matrix of *P. aeruginosa* biofilm and its regulation (Table 4). The genes for three types of exopolysaccharides (EPS), previously reported as involved in the construction of the biofilm matrix of *P. aeruginosa*, (Pel, Psl and alginate [72,75]) were found in our isolate. These EPS molecules form the protective matrix [76]. Psl is the primary factor in charge of the initiation and maintenance of the biofilm structure by providing cell to cell and cell to surface interactions [77,78,79,80]. It also works as a signaling molecule to the successive events involved in the formation of biofilms and also acts as a defensive layer for different immune and antibiotic attacks [81]. Pel polysaccharide is a glucose-rich extracellular matrix and is involved in the formation of biofilms that are attached to the solid surfaces. It is considered to be less important, compared to Psl [72,80,82,83]. In *P. aeruginosa* from clinical isolates of CF patients, alginate is produced [23]. Besides its role in maintenance and protection of biofilm structure, it is essential for water and nutrient preservation [84].

Biofilm formation is a multicellular process stimulated by environmental signals and controlled by regulatory networks. During the biofilm formation, cells undergo many phenotypic shifts that are regulated by a large array of genes [85]. In the genome of *P. aeruginosa* strain S3, several regulatory factors were identified. One of these regulatory factors was the signaling molecule bis-(3′-5′)-cyclic dimeric guanosine monophosphate (c-di-GMP), which is considered to be one of the most significant molecular determinants in biofilm regulation [86]. A c-di-GMP molecule controls the interchange between the planktonic and biofilm-associated lifestyle of bacteria by stimulating the biosynthesis of adhesins and exopolysaccharides during the formation of biofilm [87]. The bacterial cell to cell communication system known as quorum sensing (QS) is involved in the maintenance of many biological processes like biofilm formation, bioluminescence, antibiotic production, virulence factor expression, competence for DNA uptake, and sporulation [88,89]. LasR/LasI, RhlR/RhlI and PQS are the three QS signaling systems employed by *P. aeruginosa* to control biofilm formation [90,91]. These three QS signaling system were found to be the part of the genome for our isolate.

There is little information in the literature regarding the genetic elements involved in biofilm formation in *Sphingobacterium*, Genome analysis of our isolate *Sphingobacterium* sp. S2 showed the presence of some genetic elements that are reported to be involved in biofilm formation in the literature, such as genes for Stage 0 sporulation protein YaaT. This protein is reported to be involved in the sporulation process and biofilm development [92,93]. A response regulator, LuxR involved in the sensory mechanism, and gliding motility protein precursors GldC, GldJ, GldN were also detected in the genome of our strain. These proteins are known for their role in biofilm development [94]. A probable outer membrane protein precursor of OmpA family, considered to be putative adhesins and adhesion-related proteins, were also reported in *Sphingobacterium* sp. S2 [94].

*Geobacillus* is very well reported in the literature for biofilm and spore formation [95,96]. Anthranilate phosphoribosyltransferase (EC 2.4.2.18) [97] and 5′-nucleotidase (EC 3.1.3.5), reported to have an impact on virulence and biofilm formation, were found in our *Geobacillus* sp. EC-3 [98]. Cyclic-di-AMP, well known for its role in the regulation of diverse cellular pathways, including potassium homeostasis, biofilm formation, and cell wall biosynthesis, was also detected in our strain [99].

### 2.8. Enzymes

The biodegradation of polymers is carried out by two types of enzymes, extracellular enzymes that degrade long chain polymers into short oligomers or subunits that are subsequently carried inside the cell, and intracellular enzymes that further degrade the small, transported units [100,101]. The degradation of synthetic polymers in the environment can be a slow process [102,103]. PLA is a synthetic linear aliphatic polyester of lactic acid monomers joined together by ester linkages [3]. The presence of ester bonds in its backbone, make the polymer sensitive to hydrolysis, both chemically as well as enzymatically [101]. The biodegradation of polyesters is mostly carried out by esterolytic enzymes such as esterases, lipases, or proteases. In the literature, microbial degradation of PLA is reported to be conducted by proteases, lipases, esterases and a few cutinases [4]. *Sphingobacterium* spp., *P. aeruginosa*, and *Geobacillus* spp. had been documented before to have a role in the degradation of different environmental pollutants, such as mixed plastic waste, PAHs, oil, and dyes and pesticides. *P. aeruginosa* has also been reported to have the potential to degrade PLA nanocomposites [19,26,30,104,105]. In our previous studies we have reported the expression of PLA hydrolyzing lipase and esterase from our isolates *P. aeruginosa* strain S3 and *Sphingobacterium* sp. strain S2 [12,97,98]. Genome annotation of S2, S3 and EC-3 reveal the presence of numerous hydrolytic enzymes (Table 5). In the genome of *P. aeruginosa* *S3*, 75 different types of proteases, 50 esterases and 25 different types of lipases were detected (i.e., the sum of “Common to Reference Genome” and those “Unique to Strain”).

Similarly, in the genome of *Sphingobacterium* sp. S2, 36 proteases, 30 esterases and 18 lipases were identified. Of the three genomes, *Geobacillus* sp. EC-3, the smallest of the three genomes, had the most putative hydrolytic enzymes; EC-3 had 20 lipases, 127 proteases and 72 lipases.

## 3. Discussion

The present study reports the whole genome sequence analysis of three bacterial strains isolated from compost, *P. aeruginosa* S3, *Sphinogobacterium* sp. S2, and *Geobacillus* sp. EC-3, each possessing the metabolic skill of utilizing a polymeric solid, poly(lactic acid), as the sole source of carbon at ~30 °C or 58 °C. The initial steps in the degradation of solids by bacteria are extracellular, until sufficiently small molecules that can be transported across the outer cell envelope are generated through the activities of secreted enzymes. An obvious microbial strategy for optimizing the effectiveness of secreted enzymes is the migration to a position as proximal to the solid as possible, and nothing is closer than a biofilm. All three of our isolates were able to form biofilm on PLA and all three have putative biofilm markers in their genomes. Genomic analyses of *P. aeruginosa* strains have a robust literature because of its medical importance and as a consequence, there are many genetic elements that can be identified in *P. aeruginosa* S3 that are implicated in biofilm formation. These include structural elements (outer membrane matrix proteins), transport mechanisms, and regulatory elements like cyclic di-GMP and three quorum sensing systems. In addition to genetic determinants for biofilm formation, many virulence-associated genes were identified in strain S3.

Regarding *Sphingobacterium*, a recent description of a *Sphingobacterium* sp. isolate [106] capable of inhibiting the fungal activity of *Fusarium* posits that siderophores and chitinases may account for the antifungal activities and, in the genomic analysis of this strain (SJ-25), they identify potential candidate genes. Strain S2 also contains at least four siderophores as well as TonB and an abundance of TonB-dependent receptors. We also detected a chitin binding protein but no indication of excreted chitinases. Curiously, a biofilm related protein, identified as homologous to Stage 0 sporulation protein YaaT, was also detected. *Sphingobacterium* is a Gram-negative nonsporulating rod in the phylum Bacteroidetes.

Regarding *Geobacillus*, similar to *G. thermodenitrificans* T12 [107], there is a respiratory nitrate reductase (alpha, beta, gamma, and delta subunits) as well as CRISPR elements. In addition, UDP-glucose 4-epimerase and mannose-1-phosphate guanylyltransferase, identified in *Geobacillus* sp. WSUCF1 [108], were also detected in strain EC-3.

Draft genomes of strains S2, S3 and EC-3 were studied to gain an insight into the genetic elements that are involved in the degradation of PLA. The catabolic genes responsible for biodegradation of different xenobiotic compounds, genes responsible for formation and regulation of biofilm, genes for transport and utilization of lactate and several enzymes predicted to be involved in the degradation of many organic pollutants were identified. Of interest was the apparent lack of lactate permease in *Sphingobacterium* sp. S2, suggesting that an alternative mode of attack on PLA leads to a molecule other than lactate, that is transportable, or that lactate is further modified extracellularly before transport. Finally, Chai, et al. [109] have detected catabolic lactate genes, lutA, lutB and lutC, in *Bacillus subtilis* and note that the upregulation of these genes is required for growth on lactate as the sole carbon source. Moreover, these genes are under dual regulation, permitting expression in liquid culture as well as within a biofilm. This gene cluster was also found in *Geobacillus* sp. EC-3. Common features of these three phylogenetically disparate isolates were an abundance of genetic determinants for hydrolytic enzymes and biofilm formation. These three strains were isolated through serial selection on a single carbon source, PLA. The genomic analyses provide insights into the potential array of genes that may be required for the efficient degradation of PLA.

## 4. Materials and Methods

### 4.1. Strains and Media

Strains were grown routinely on R2A agar (Difco) or R2broth (Yeast extract 0.5 g/L, Proteose Peptone No. 3 0.5 g/L, Casamino Acids 0.5 g/L, Dextrose 0.5 g/L, Soluble Starch 0.5 g/L, Sodium Pyruvate 0.3 g/L, Dipotassium Phosphate 0.3 g/L, Magnesium Sulfate 0.05 g/L). Strains were stored at −80 °C in 20% glycerol and streaked onto R2A to initiate regrowth. All chemicals and media were purchased from Sigma-Aldrich, Inc., Saint Louis, MO, USA.

### 4.2. DNA Extraction

Three of our previously isolated PLA degrading bacterial strains, *Sphingobacterium* sp. strain S2, *P. aeruginosa* strain S3 and *Geobacillus* EC-3 (GenBank accession numbers *KY432687*, *KY432688*, and *MH183212*, respectively) were selected for genome sequencing [12]. Strains S2 and S3 were grown separately in 100 mL of LB in a 250 mL Erlenmeyer flask for 16 h in a shaking incubator at 30 °C and 70 rpm. *Geobacillus* EC-3 was grown in M9 minimal media shaking at 58 °C. Cells were pelleted (10,000 RPM in a Sorvall SS34 rotor, Beckman Coulter Life Sciences, Indianapolis, Indiana USA) and genomic DNA was isolated using MO BIO PowerSoil DNA isolation kit (MO BIO laboratories, Inc., Loker Ave West, Carlsbad, CA, USA). NanoDrop ND-1000 spectrophotometer and ND-1000 V3.1.8 software (Thermo Fisher Scientific Inc., Wilmington, DE, USA) were used to determine DNA concentrations of purified samples and sent for whole genome sequencing at Michigan State University Genomics Facility (MSU-RTSF), East Lansing, MI, USA.

### 4.3. Genome Sequencing

Libraries for sequencing were prepared using the Illumina TruSeq Nano DNA Library Preparation Kit (Illumina, Inc., San Diego CA, USA) on a Perkin Elmer Sciclone NGS robot (Perkin Elmer, Boston, MA, USA). Before sequencing, the qualities of the libraries were tested and quantification was performed using a combination of Qubit dsDNA HS (Thermo Fisher Scientific, Waltham, MA, USA), Caliper LabChip GX HS DNA (Perkin Elmer, Boston, MA, USA) and Kapa Illumina Library Quantification qPCR assays (Roche Sequencing and Life Science, Wilmington, MA, USA). Libraries were pooled in equimolar quantities and loaded on an Illumina MiSeq standard v2 flow cell with a 2 × 250 bp paired end format and using a v2 500 cycle reagent cartridge. Illumina Real Time Analysis (v1.18.64) was used for base calling and the output was converted to FastQ format with Illumina Bc12fastq (v1.8.4) after demultiplexing (Illumina, Inc., San Diego CA, USA). A total of 6,304,420 reads were obtained for strain S2, 5,800,229 reads for strain S3 and 5,730,761 reads for strain EC-3. The genomic reads have been deposited at the Sequence Read Archives at NCBI with Bioproject IDs of SRP149807 and PRJNA721072.

### 4.4. Sequence Assembly, Annotation, and Analysis

Assembly of the genomes was performed using the full Spades assembly function within PATRIC (PATRIC 3.4.9) [34] as implemented in the *miseq* assembly option. This assembly option incorporates BayesHammer algorithms followed by Spades, (Spades version 3.8.). Rast tool kit as implemented in PATRIC [110] was used for the annotation of contigs. The assembled contig file generated from this assembly was used as seed for the Comprehensive Genome Analysis function in PATRIC. The genomes were interrogated for the distribution of specific protein families (PGFams) using the protein family sorter tool on PATRIC. The genomes were compared to their closest reference genomes available on PATRIC to examine the strain-specific unique proteins as well as proteins common to the closest relative using the filter option in protein family sorter tool on PATRIC.

### 4.5. Average Nucleotide Identity (ANI) for Species Delineation

Isolates were further analyzed using a whole genome based average nucleotide identity (ANI) method to delineate the genomes to their correctly matching relatives. ANI values were calculated using MiSI (microbial species identifier) tool that is publicly available at the Integrated Microbial Genomes (IMG) database [111]. The algorithm used in the original method proposed by Konstantinidis and Tiedje was modified and used to determine ANI between two genomes [112]. The average of the nucleotide identity of the orthologous genes of the pair of genomes was calculated and identified as bidirectional best hits (BBHs) using a similarity search tool accessed in January, 2019, NSimScan (http://www.scidm.org/, accessed on 7 June 2021). The ANI of one genome to another genome is defined as the sum of the %-identity times the alignment length for all best bidirectional hits, divided by the sum of the lengths of the BBH genes. This pairwise calculation is performed in both directions. The strains used for comparison were complete genomes obtained from NCBI and are as follows. For the *Sphingobacterium* comparisons; *S. thalpophilum* DSM 11723 (Draft genome of 32 contigs), *S.* sp. G1-14, S. sp. B29, *S. multivorum* DSM 11691, *S. lactis* DSM 22361, *S. wenxiniae* DSM 22789, *S. mizutaii* DSM 11724 and *S. sp*. 21. For the *Pseudomonas aeruginosa* comparisons; *P. aeruginosa* PSE302, *P. aeruginosa* PA96, *P. aeruginosa* PA01H20, *P. aeruginosa* DSM 50071 *P. aeruginosa* PAO1, *P. aeruginosa* PAK, *P. aeruginosa* O12 PA7, *P. aeruginosa* PA_D25, *P. aeruginosa* PA_D1, *P. aeruginosa* KU and *P. aeruginosa* T52373. Regarding *Geobacillus* sp. comparison; EC-3 *Geobacillus thermoleovorans* CCB_US3_UF5, *Geobacillus thermoleovorans* strain SGAir0734, *Geobacillus thermocatenulatus* strain BGSC 93A1, *Geobacillus* sp. A8 and *Geobacillus kaustophilus* NBRC 102445 were used.

### 4.6. Comparative Alignments Using MeDuSa and MAUVE

For the comparative alignment of the genomes with their reference genomes and their visualization, MeDuSa [41] was used to reduce the number of contigs through comparison with the gene order of the closest strain. This was followed by alignment with MAUVE [42] to a reference strain to provide an estimate of alignment similarity. The reference strain selected was the closest finished genome in the public database. Alignments in MAUVE were also performed with contigs directly from the Spades assembly. *P. aeruginosa* PSE305 was used as a reference for strain S3 (ANI of 98.22) while *Sphingobacterium thalpophilum* NCTC11429 (ANI of 98.46) was selected as a reference for *Sphingobacterium sp.* S2. According to the “Similar Genome Finder” function implemented in “PATRIC”, *G. thermoleovorans* strains B23 and CCB_US3_UF5 from public databases were closest to EC-3. This function uses *Mash*/*MinHash* [113] to determine genome similarities. Strain CCB_US3_UF5 was used as the reference strain in MAUVE as this genome has been finished (1 chromosome) and has an ANI similarity of 99.9%.

## Figures and Tables

**Table 1:** General genomic features of *Sphingobacterium* S2, *P. aeruginosa* S3 and *Geobacillus* EC-3.

Features	*Pseudomonas* S3	*Sphingobacterium* S2	*Geobacillus* EC-3	Database ^1^
Contigs	63	87	111	PATRIC
G + C Content (%)	66.26	43.66	52.18	PATRIC
Plasmids	ND ^2^	ND	ND	PATRIC
Contig L50	8	9	12	PATRIC
Genome Length	6,509,961 bp	5,445,390 bp	3,397,712 bp	PATRIC
Contig N50	273,159 bp	267,833 bp	90,542 bp	PATRIC
Longest Contig	658,980 bp	434,944 bp	321,190 bp	PATRIC
Chromosomes	ND2	ND	ND	PATRIC
CDS	6239	4951	3790	PATRIC
tRNA	60	72	87	PATRIC
Repeat Regions	60	0	138	PATRIC
rRNA	7	3	11	PATRIC
Hypothetical proteins	1307	2087	984	PATRIC
Proteins with functional assignments	4932	2864	2806	PATRIC
Proteins with EC number assignments	1287	933	989	PATRIC
Proteins with GO assignments	1091	806	750	PATRIC
Proteins with Pathway assignments	970	693	678	PATRIC
Antibiotic Resistance	51	0	0	CARD
Antibiotic Resistance	5	0	0	NDARO
Antibiotic Resistance	100	28	32	PATRIC
Drug Target	67	0	25	DrugBank
Drug Target	10	0	1	TTD
Transporter	186	2	18	TCDB
Virulence Factor	1	0	2	PATRIC_VF
Virulence Factor	233	0	0	VFDB
Virulence Factor	86	0	2	Victors

^1^ Database used for identifications. ^2^ Not Determined.

**Table 2:** Pathways and number of genes involved in aromatic compound metabolism in draft genome sequences of *P. aeruginosa* S3, *Sphingobacterium* sp. S2 and *Geobacillus* sp. EC-3. Unique EC count refers to the number of unique enzymes identified.

	*P. aeruginosa* S3		*Sphingobacterium* sp. S2		*Geobacillus* sp. EC-3	
Pathway Name	Unique Gene Count	Unique EC Count	Unique Gene Count	Unique EC Count	Unique Gene Count	Unique EC Count
1- and 2-Methylnaphthalene degradation	20	8	8	6	3	1
1,4-Dichlorobenzene degradation	43	13	18	6	17	6
2,4-Dichlorobenzoate degradation	17	9	7	3	5	3
Atrazine degradation	10	2	7	2	5	2
Benzoate degradation via hydroxylation	60	25	16	7	19	6
Biphenyl degradation	3	2	3	2	0	0
Bisphenol A degradation	13	6	4	3	1	1
Caprolactam degradation	21	6	9	4	12	4
Drug metabolism-cytochrome P450	19	3	3	2	3	1
Drug metabolism-other enzymes	9	9	8	8	11	10
Ethylbenzene degradation	10	3	6	3	6	2
Fluorobenzoate degradation	9	7	1	1	1	1
γ-Hexachlorocyclohexane degradation	9	6	2	2	0	0
Geraniol degradation	30	9	9	4	16	4
Naphthalene and anthracene degradation	16	7	6	3	4	2
Styrene degradation	14	7	2	2	3	2
Tetrachloroethene degradation	29	8	6	4	8	2
Toluene and xylene degradation	14	6		2	0	0
Trinitrotoluene degradation	12	4	10	3	2	2
DDT degradation	5	4	2	2	0	0
Metabolism of xenobiotics by cytochrome P450	19	3	5	3	3	1
Styrene degradation	0	0	0	0	3	2
Fluorobenzoate degradation	0	0	0	0	1	1

**Table 3:** Genes for lactate metabolism in *P. aeruginosa* S3, *Sphingobacterium* sp. S2 and *Geobacillus* sp. EC-3.

**Proteins for Lactate Utilization in *Pseudomonas aeruginosa* S3**		
**Description**	**AA Length**	**Proteins**
Acetolactate synthase large subunit (EC 2.2.1.6)	574	1
Acetolactate synthase small subunit (EC 2.2.1.6)	163	2
d-lactate dehydratase (EC 4.2.1.130)	291	1
d-lactate dehydrogenase (EC 1.1.1.28)	329	1
l-lactate dehydrogenase	383	2
l-lactate permease	560	1
Lactate-responsive regulator LldR, GntR family	257	1
Predicted d-lactate dehydrog., Fe-S protein, FAD/FMN-containing	938	1
**Proteins for Lactate Utilization in *Sphingobacterium* sp. S2**		
**Description**	**AA Length**	**Proteins**
Acetolactate synthase large subunit (EC 2.2.1.6)	606	1
Acetolactate synthase small subunit (EC 2.2.1.6)	196	1
d-lactate dehydratase (EC 4.2.1.130)	145	2
d-lactate dehydrogenase (EC 1.1.1.28)	330	1
Fe-S protein, homolog of lactate dehydrogenase SO1521	974	1
l-lactate dehydrogenase	389	1
Predicted l-lactate dehydrogenase, Fe-S oxidoreductase subunit YkgE	242	1
Predicted l-lactate dehydrogenase, hypothetical protein subunit YkgG	215	1
Predicted l-lactate dehydrogenase, Iron-sulfur cluster-binding subunit YkgF	462	1
**Proteins for Lactate Utilization in *Geobacillus* sp. EC-3**		
**Description**	**AA length**	**Proteins**
l-lactate dehydrogenase (EC 1.1.1.27)	317	1
l-lactate permease	557	1
Lactate utilization protein LutA	239	1
Lactate utilization protein LutB	476	1
Lactate utilization protein LutC	240	1
Lactate-responsive regulator LutR, GntR family	241	1
Probable 2-phosphosulfolactate phosphatase (EC 3.1.3.71)	260	1

**Table 4:** Genetic elements involved in biofilm formation and regulation detected in P. aeruginosa S3, Sphingobacterium S2 and Geobacillus EC-3.

Factors involved in Biofilm formation and regulation detected in *P. aeruginosa* S3	
**Extracellular Matrix Components**	***Pseudomonas* Quinolone Signal (PQS)**
Extracellular Matrix protein PslA	PQS biosynthesis protein PqsH, similar to FAD-dependent monooxygenases
Extracellular Matrix protein PslC	PQS biosynthesis protein PqsA, anthranilate-CoA ligase (EC 6.2.1.32)
Extracellular Matrix protein PslD	PQS biosynthesis protein PqsB, similar to 3-oxoacyl-[acyl-carrier-protein] synthase III
Extracellular Matrix protein PslE	PQS biosynthesis protein PqsC, similar to 3-oxoacyl-[acyl-carrier-protein] synthase III
Extracellular Matrix protein PslF	PQS biosynthesis protein PqsD, similar to 3-oxoacyl-[acyl-carrier-protein] synthase III
Extracellular Matrix protein PslG	PqsE, quinolone signal response protein
Extracellular Matrix protein PslL	Anthranilate synthase, aminase component (EC 4.1.3.27)
Extracellular Matrix protein PslJ	Anthranilate synthase, amidotransferase component (EC 4.1.3.27)
Extracellular Matrix protein PslK	Multiple virulence factor regulator MvfR/PqsR
Extracellular Matrix protein PelG	Putative transcriptional regulator near PqsH
Extracellular matrix protein PelF, glycosyltransferase, group 1	**c-di-GMP**
Extracellular Matrix protein PelE	3’,5’-cyclic-nucleotide phosphodiesterase (EC 3.1.4.17)
Extracellular Matrix protein PelD	5’-nucleotidase/2’,3’-cyclic phosphodiesterase and related esterases
Extracellular Matrix protein PelC	Acyl carrier protein phosphodiesterase (EC 3.1.4.14)
Extracellular Matrix protein PelB	diguanylate cyclase/phosphodiesterase (GGDEF & EAL domains)
Extracellular Matrix protein PelA	Glycerophosphoryl diester phosphodiesterase (EC 3.1.4.46)
Alginate regulatory protein AlgQ	Phosphodiesterase/alkaline phosphatase D
Alginate regulatory protein AlgP	Membrane bound c-di-GMP receptor LapD
Alginate biosynthesis protein AlgZ/FimS	**Quorum sensing systems**
Alginate biosynthesis transcriptional regulatory protein algB	N-3-oxododecanoyl-L-homoserine lactone quorum-sensing transcriptional activator
Alginate biosynthesis protein Alg8	regulator LasR & regulator RhlR
Alginate biosynthesis protein Alg44	N-acyl-L-homoserine lactone synthetase LasI
Alginate biosynthesis protein AlgK precursor	N-butyryl-L-homoserine lactone quorum-sensing transcriptional activator
Outer membrane protein AlgE	**Factors involved in Biofilm formation and regulation detected in *Sphingobacterium* sp. S2**
Alginate biosynthesis protein AlgX	Anthranilate phosphoribosyltransferase (EC 2.4.2.18)
Alginate lyase precursor (EC 4.2.2.3)	Anthranilate synthase, aminase component (EC 4.1.3.27)
Alginate biosynthesis protein AlgJ	Carboxymuconolactone decarboxylase (EC 4.1.1.44)
Alginate o-acetyltransferase AlgF	5’-nucleotidase (EC 3.1.3.5)
Alginate biosynthesis transcriptional activator	Acyl carrier protein
**Factors involved in Biofilm formation and regulation detected in *Geobacillus* sp. EC-3**	Transciptional regulators LysR family & MarR/EmrR family
2’,3’-cyclic-nucleotide 2’-phosphodiesterase, Bsub YmdB	DNA binding response regulator, LuxR family
5’-nucleotidase (EC 3.1.3.5)	Sugar transferase
5’-nucleotidase family protein in cluster with NagD-like phosphatase	Polysaccharide biosynthesis protein
Acyl carrier protein	Two-component transciptioal response regulator, RprY, OmpR family
Anthranilate phosphoribosyltransferase (EC 2.4.2.18)	Gliding motility protein precursor GldC, GldJ & GldN
Anthranilate synthase, aminase component (EC 4.1.3.27)	OmpA domain protein
Biofilm-associated protein	OmpA family protein
Carboxymuconolactone decarboxylase (EC 4.1.1.44)	OmpA/MotB
Cyclic-di-AMP phosphodiesterase GdpP	Glycerophosphoryl diester phosphodiesterase (EC 3.1.4.46)
Glycerophosphoryl diester phosphodiesterase (EC 3.1.4.46)	Stage 0 sporulation protein YaaT
Glycerophosphoryl diester phosphodiesterase (EC 3.1.4.46) periplasmic (secreted in GramPositives)	
Lactate Utilization Protein A	
Lactate Utilization Protein B	
Lactate Utilization Protein C	

**Table 5:** Different type of hydrolytic enzymes found in the draft genome of *P. aeruginosa* S3, *Sphingobacterium* sp. S2, and *Geobacillus* sp. EC-3.

	*P. aeruginosa* S3		*Sphingobacterium* sp. S2		*Geobacillus* sp. EC-3	
Enzyme	Common to Reference Genomes	Unique to Strain S3	Common to Reference Genomes	Unique to Strain S2	Common to Reference Genomes	Unique to Strain EC-3
Hydrolase	123	51	84	8	173	54
Lipase	20	5	10	8	15	5
Protease	57	18	22	14	91	36
Esterase	33	17	25	5	53	19
Phospho-diesterase	8	5	5	4	16	4
Oxygenase	63	19	7	3	45	9
Catalase	6	6	2	1	5	3
Phosphatase	66	17	25	13	77	33
Common + Unique		514		236		638

## Data Availability

Sequences for the three genomes have been deposited at the Sequence Read Archive at NCBI with the accession numbers SRP149807 and PRJNA721072.

## Supplementary Materials

The following is available online at https://www.mdpi.com/article/10.3390/ijms22147385/s1, Figure S1: MAUVE alignment of five *Geobacillus* genomes.
