# Supplementary material for: Genome Annotation of Poly(lactic acid) Degrading Pseudomonas aeruginosa, Sphingobacterium sp. and Geobacillus sp
Source: Int J Mol Sci. 2021 Jul 10;22(14):7385. doi: 10.3390/ijms22147385 (PMC8305213; doi:10.3390/ijms22147385)
Supplement: Supplementary file 1 [file ijms-22-07385-s001.zip › ijms-1273164-supplementary.pdf]

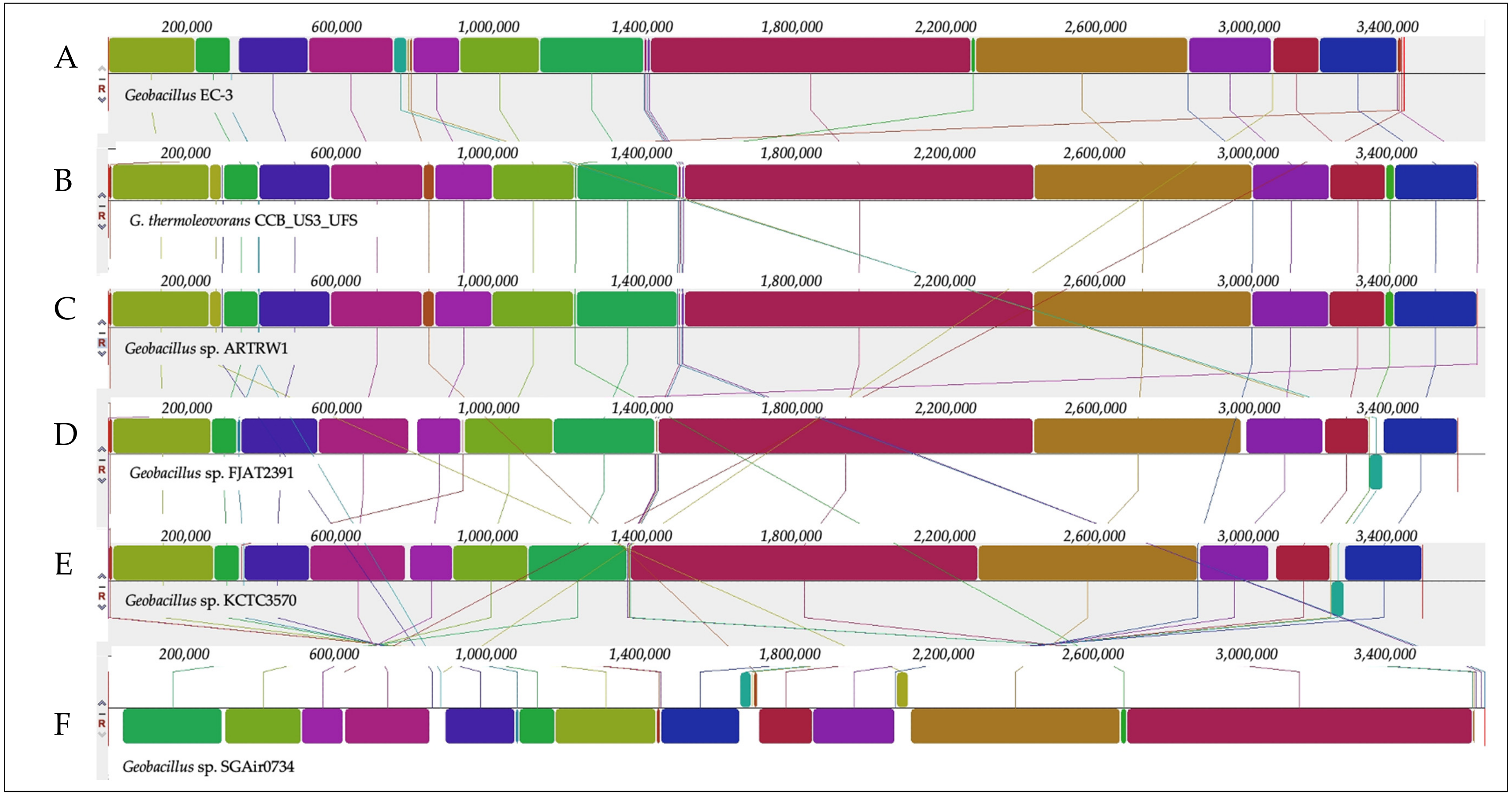

Figure S1. MAUVE alignment of six *Geobacillus* genomes. A, *Geobacillus* EC-3 (Genbank PRJNA721072); B, *G. thermoleovorans* CCB_US3_UFS (Genbank NC_016593); C, *G. thermoleovorans* ARTRW1 (Genbank NZ_CP042251); D, *Geobacillus* FJAT-2391 (Genbank CP017071); E, *G. thermoleovorans* KCTC 3570 (Genbank CP014335); F, *G. thermoleovorans* SGAir0734 (Genbank NZ_CP027303).
